# New Insights Into the Role of Mitochondria Quality Control in Ischemic Heart Disease

**DOI:** 10.3389/fcvm.2021.774619

**Published:** 2021-11-26

**Authors:** Yanguo Xin, Xiaodong Zhang, Jingye Li, Hui Gao, Jiayu Li, Junli Li, Wenyu Hu, Hongwei Li

**Affiliations:** ^1^Department of Cardiology, Cardiovascular Center, Beijing Friendship Hospital, Capital Medical University, Beijing, China; ^2^General Surgery Department, Beijing Friendship Hospital, Capital Medical University, Beijing, China; ^3^National Clinical Research Center for Digestive Diseases, Beijing, China; ^4^Laboratory of Heart Valve Disease, West China Hospital, Sichuan University, Chengdu, China; ^5^Department of Cardiology, The First Affiliated Hospital of China Medical University, Shenyang, China; ^6^Beijing Key Laboratory of Metabolic Disorder Related Cardiovascular Disease, Beijing, China; ^7^Department of Geriatrics, Cardiovascular Center, Beijing Friendship Hospital, Capital Medical University, Beijing, China

**Keywords:** mitochondria, myocardial infarction, metabolism, remodelling, inflammation

## Abstract

IHD is a significant cause of mortality and morbidity worldwide. In the acute phase, it's demonstrated as myocardial infarction and ischemia-reperfusion injury, while in the chronic stage, the ischemic heart is mainly characterised by adverse myocardial remodelling. Although interventions such as thrombolysis and percutaneous coronary intervention could reduce the death risk of these patients, the underlying cellular and molecular mechanisms need more exploration. Mitochondria are crucial to maintain the physiological function of the heart. During IHD, mitochondrial dysfunction results in the pathogenesis of ischemic heart disease. Ischemia drives mitochondrial damage not only due to energy deprivation, but also to other aspects such as mitochondrial dynamics, mitochondria-related inflammation, etc. Given the critical roles of mitochondrial quality control in the pathological process of ischemic heart disease, in this review, we will summarise the efforts in targeting mitochondria (such as mitophagy, mtROS, and mitochondria-related inflammation) on IHD. In addition, we will briefly revisit the emerging therapeutic targets in this field.

## Introduction

**Figure 1:**
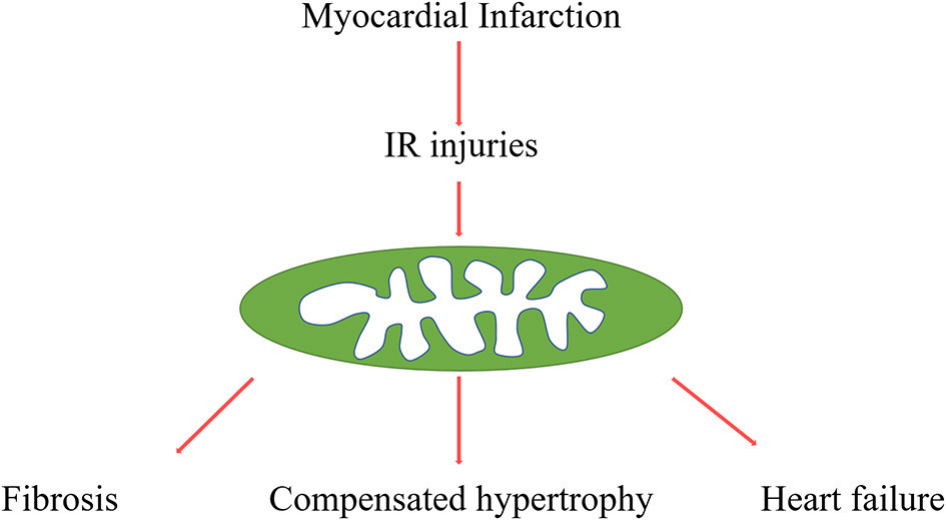
Procedures from myocardial infarction to post-infarction remodelling. Myocardial infarction and reperfusion attributed mitochondria damage, mitochondria function, and structure disorders are involved in various pathophysiological processes, such as cardiac fibrosis, compensated hypertrophy, and final heart failure.

AMI is myocardial necrosis due to acute obstruction of a coronary artery or induced hypoperfusion of myocardial tissue, which is attributed to millions of deaths worldwide every year. The longer duration of ischemia usually leads to much more severe myocardial damage. Therefore, timely reestablishment of blood flow is the critical factor for rescuing the ischemic tissue. However, another form of strike, called IRI is also responsible for local damage (1, 2) (Figure 1). According to previous evidence, IRI describes tissue ischemia with inadequate oxygen supply after successful reperfusion of the culprit artery. Currently, there are no efficient strategies to prevent the damage caused by IRI (3). Thus, more explorations of the underlying molecular mechanisms are urgent to foster the identification of novel agents to improve outcomes following MI or IRI.

Adverse myocardial remodelling is a significant feature of acute myocardial infarction, characterised by various gradual changes of left ventricular morphology, such as infarcted zone expansion and chamber dilatation. HF is the end stage of myocardial remodelling after AMI. It's diagnosed in ~13% of patients at 30 days and 20–30% at 1 year after discharge for MI (4, 5) (Figure 1). In addition, accumulated evidence indicated that myocardial inflammation and myocardial fibroblasts play critical roles in the process of cardiac repairment after AMI, but excessive inflammation and fibrosis also lead to cardiac remodelling (6, 7). Cardiac fibrosis has been an independent risk factor in HF, which attributed HF patients to sudden cardiac death and increased overall mortality independently of the ejection fraction (8). Although various studies focus on the underlying mechanisms of this pathological process, there are still many unsolved problems in this field, and there are no effective strategies to reverse this process.

Mitochondria is the energy house of cardiomyocytes, generating ATP to maintain normal heart contractile function (9). Mitochondria metabolic dysfunction is a key characteristic of ischemic heart disease. In addition, with more studies focusing on mitochondria, it's reported that mitochondria is not only an energy organelle, but also closely connected with apoptosis (10), ROS generation (11–13), lipid metabolism (14–16), and inflammation (17). All these mechanisms contribute to acute phase and post-infarction remodelling (18). In this review, we discuss the roles of mitochondria in the pathological of ischemic heart disease and the potential in translating mito-protective strategies into the clinical setting.

## Mitochondrial Remodelling in Ischemic Heart Disease

**Figure 2:**
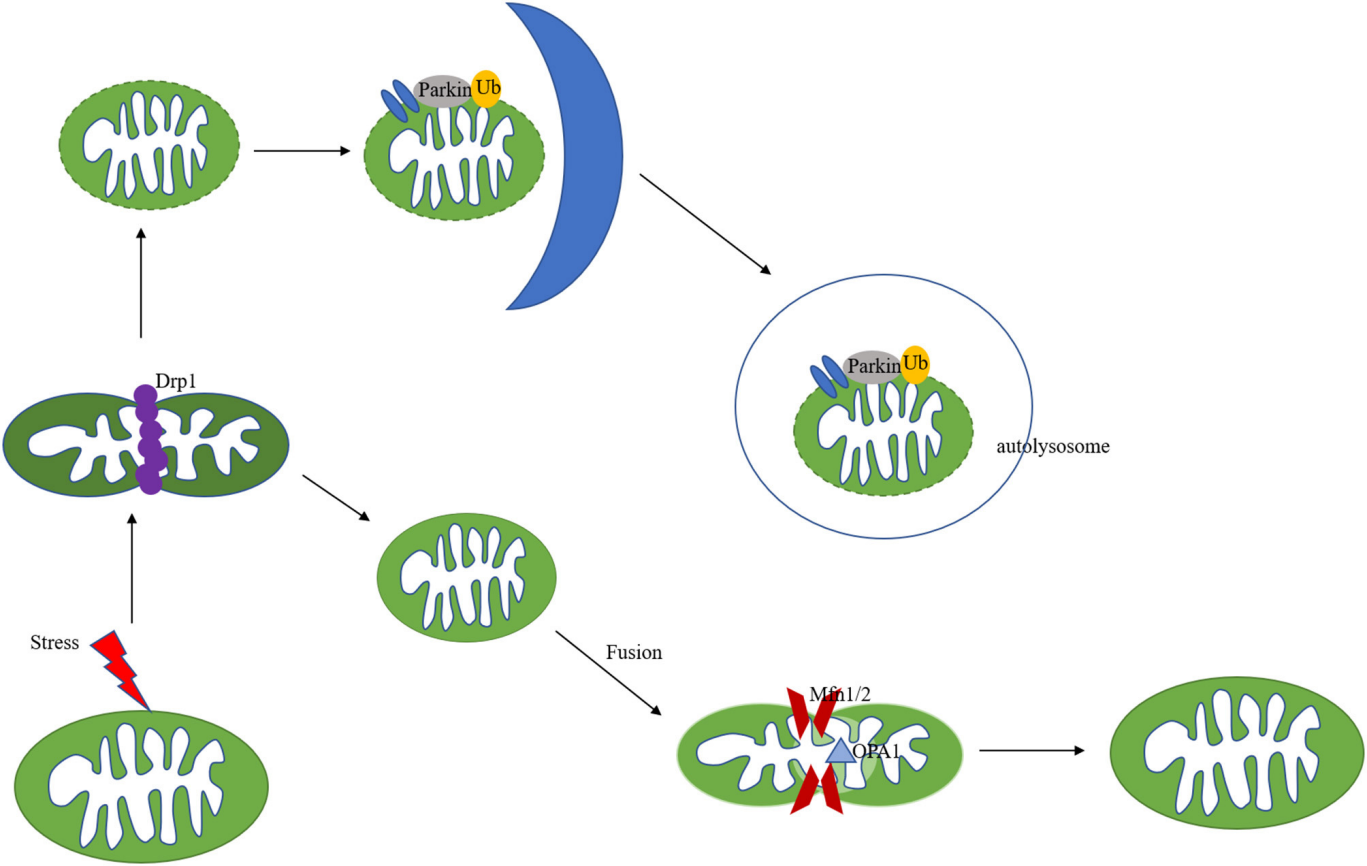
Mechanisms of mitochondria dynamics and mitophagy. Mitochondria are highly dynamic organelles undergoing coordinated cycles of fission and fusion. A series of GTPase-related proteins are involved in the dynamic process. Drp1 is the main regulator of mitochondrial fission, and Mfn1/2, combined with OPA1 regulate the fusion process. After mitochondrial fission, the mitochondrial fragments could be cleared out via PINK/Parkin-mediated mitophagy pathway.

Mitochondrial remodelling in ischemic heart disease includes structural and metabolic changes, both of which are identified to play key roles through each stage of the pathogenesis of ischemic heart disease. In cardiomyocytes, mitochondria are highly dynamic organelles, in response to environmental or metabolic changes, they underwent continuous fission, fusion and cristae remodelling (Figure 2). Fusion is an essential dynamic process to maintain the equilibration of matrix metabolites, intact mtDNA, and even membrane components (19–21). In reverse, mitochondrial fission exerts the function to segregate dysfunctional mitochondria to clean damaged proteins and mtDNA (19, 22). These mitochondrial activities are strictly regulated by a group of GTPases related to the dynamin family (23). Through the control of these proteins, mitochondria can maintain a dynamic fission-fusion balance to exert physiological functions. The structural disequilibrium is closely related to acute and chronic heart diseases, involving various molecular mechanisms (discussed in the following parts).

**Figure 3:**
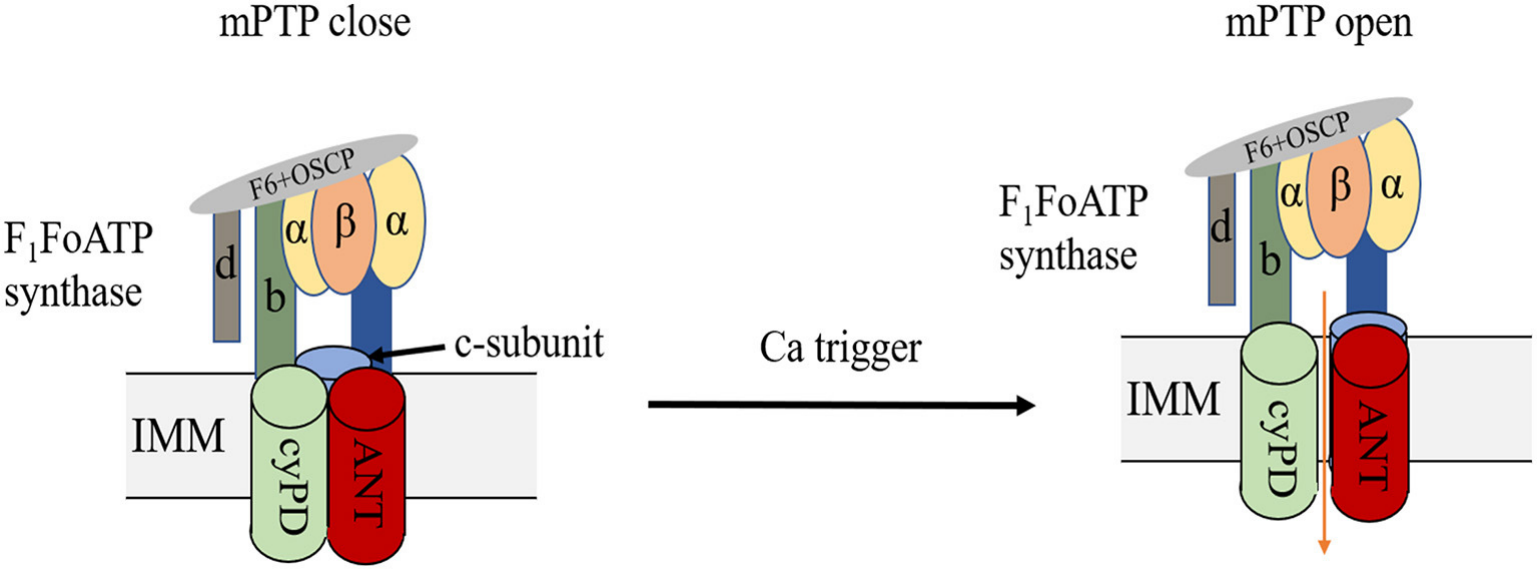
Structure of mPTP. mPTP is a non-specific and -selective channel composed of multiple proteins, which is voltage-dependent and spans cytoplasm, OMM, IMM, and mitochondrial matrix. F_1_F_0_ (F)-ATP synthase is the main component of the pore and that the regulatory molecule CypD is a protein modulator of the mPTP.

The double-membrane mitochondria mediate OXPHOS, coupling the substrate oxidation to ATP generation, which is also known as the electron transport chain, ETC (24, 25). Mitochondrial energy remodelling is a key characteristic of myocardial ischemia. In the early ischemia stage, FAO, the main metabolic way of heart, increases slightly and provides 60–90% of cardiac ATP production. In addition, the rapid depletion of oxygen switches mitochondria metabolism to glycolysis (26), resulting in pyruvate and lactate accumulation, followed by intracellular acidification. After reperfusion, the restoration of oxygen may initiate the burst of ROS, resulting in severe intracellular damage. ROS, combined with calcium overload, will trigger the opening of the mPTP. Despite various researches on this topic, the exact molecular composition of mPTP is still controversial (Figure 3). Previous evidence indicated that mPTP includes ANT, VDAC, CyPD and PiC (27, 28), but genetic ablation of these proteins revealed that they are the regulators but not the pore of mPT (29, 30). Mitochondrial F_1_F_0_ ATP synthase is known to form dimers in the inner mitochondrial membrane (31, 32). Some studies indicated that the ablation of the main membrane-embedded component of ATP synthase, c-subunit, resulted in no change of the sensitivity of mPT (33, 34), however, other evidence found that c-subunit knockout lead to attenuate mPT (35, 36). Previous studies found that mitochondrial F_1_F_0_ ATP synthase dimers were essential to form the inner mitochondrial membrane channel, maintaining their physical function (37, 38). However, Nelli et al. found that ATP synthase monomer is sufficient, and dimer formation is not required, for mPTP activity (39). Anyway, despite the controversial components of mPTP, current evidence indicated that mPTP opening could lead to the depolarization of mitochondrial membrane potential followed by cell death. Upon heart failure, FAO and mitochondrial OXPHOS decrease, resulting in cardiac ATP behind the requirement. Although the slight increase of glucose uptake and glycolysis could exert a compensatory response, this upregulation is insufficient to restore ATP production (40–42). Accumulating evidence suggest that mitochondrial respiration disturbance is a potential contributory factor to ischemic heart disease due to its generation of ROS (43). Mitochondrial remodelling participates in many regulating processes in ischemic heart disease, which will be discussed in the following parts.

## Mitochondria Mitophagy in Ischemic Heart Disease

Mitophagy is a specific subtype of autophagy, which is also an important mitochondria quality control system to maintain mitochondrial homeostasis (44, 45). The mechanisms of mitophagy induced by mitochondrial stress are complicated (Figure 2). Mitophagy impairment causes the accumulation of defective organelles, leading to cell and tissue damage. Previous studies focused on autophagosome-mediated mitophagy via LC3 adapters. Recently, many studies have investigated a PINK/Parkin pathway involved in mitophagy. PINK and Parkin were first reported as genetic factors of Parkinson's disease (46). PINK is a mitochondrial serine/threonine-protein kinase and Parkin is a cytosolic E3-ubiquitin ligase (47, 48). Under physical conditions, PINK is transported to the inner mitochondrial membrane and cleaved by MPP. The auto-phosphorylation of PINK recruited Parkin translocation to the mitochondrial surface (49, 50). Pathological stresses cause mitochondrial membrane depolarization and reduce the cleavage of PINK. Accumulated PINK could be self-phosphorylated and activated, recruiting Parkin to damaged mitochondria and triggering its E3 ligase activity (51, 52). PINK could also phosphorylate ubiquitin (Ub), forming poly-Ub chains on dysfunctional mitochondria. Parkin would be activated by PINK after binding with phospho-Ub, amplifying mitophagy signals (53). Parkin could polyubiquitinate its substrates, such as VDAC1 and Mfn1/2, leading to their degradation by the proteasomes (54, 55), followed by mitochondria fission and mitophagy (Figure 2). PINK-Parkin pathway also interferes with other mitochondrial quality control mechanisms, mitochondrial fusion, and fission play an essential role in mitophagy, mediating by dynamics-related proteins (the fission and fusion proteins) (56). Mitochondrial fission results in small fragmented mitochondria while fusion forms the elongated interconnected network. Mitochondria are divided into polarised and depolarised daughter mitochondria. Mitochondrial fission is usually considered as the prerequisite for the occurrence of mitophagy. Drp1 knockout disrupts mitochondria fission, promotes elongated mitochondria, and inhibits mitophagy, which aggravates cardiac dysfunction during IR injury (57). Fission inhibition resulted in the progression of cardiac injury due to impaired mitophagy, in addition, overexpression of Drp1 could promote mitophagy-mediated cell death (58, 59). Song et al. reported that Drp1 ablation interrupts mitochondrial fission and increases the activation of Parkin-mediated mitophagy, and Parkin deletion in Drp1-knockout mice rescues heart function and alleviates cardiac remodelling (60). NR4A1 could aggravate IR injury via increasing mitochondrial fission through Drp1 translocation and mitophagy suppression, NR4A1 ablation could protect against pathological fission and mitochondrial dysfunction. Novel therapeutic targeting the balance among NR4A1, fission, and mitophagy may improve cardiac function following IR injury (61).

In addition to increased mitochondrial fission during I/R injury, the decreased mitochondrial fusion promoted mitochondrial fragmentation, resulting in cardiac cell death and dysfunction. Mitochondrial fusion, mediated by mitofusin 1/2 and OPA1, could prevent damaged mitochondria from fusing with healthy ones. The dynamin-related protein OPA1, located on the inner mitochondrial membrane, protects against apoptosis by preventing the release of cytochrome c from the mitochondria (62). Chen et al. found that OPA1 decreased in samples from human hearts with ischemic cardiomyopathy (63). Increased ROS reduced the expression of OPA1 and aggravated cardiomyocytes apoptosis in response to I/R injury (64). OPA1 overexpression protected cardiomyocytes against hypoxia-induced damage and enhanced cell viability by inducing mitophagy (65), and melatonin could attenuate IRI via improving mitophagy and activating the AMPK-OPA1 signalling pathway (66). Additionally, Lichun et al. (67) found that increased expression of MCU induced calpain activation, down-regulating OPA1 and leading to myocardial IRI.

Current evidence indicated that impaired mitophagy participated in cardiac IRI. PTENα deficiency could disrupt mitophagy and lead to the accumulation of damaged mitochondria, followed by the higher risk of IR injury (68). WDR26 is a scaffolding protein that was found to increase after cardiac ischemia. Increasing the expression of WDR26 could increase mitochondria potential, thereby inhibit cardiomyocyte apoptosis via promoting Parkin-mediated mitophagy (69). Many agents such as antioxidants from grapes were reported to exert protection against IR injury by promoting the PINK/Parkin pathway (70, 71). Zinc ion also demonstrated cardiac protection from IR injury via promoting PINK-dependent mitophagy through the MAPK/ERK pathway, the activation of PINK/Parkin-dependent mitophagy could significantly decrease mitochondrial superoxide generation and oxidative stress (72). Cardiac Drp1 heterozygous knockout mice suffer disturbed mitophagy and are more susceptible to IR injury (57). FUNDC1 is a mitophagy receptor after hypoxia (73), exerting a protective property in cardiac IR injury. A decrease of FUNDC1 could increase ROS levels and promote apoptosis, leading to an increase in cardiac IR injury via MAPK/ERK-CREB pathway. Restoration of FUNDC1 levels could reduce myocardial infarct size (74). In addition, platelet activation and thrombosis formation is the key step in cardiac ischemia (75). Platelet-specific FUNDC1 ablation induces worse cardiac damage via mitophagy interruption and platelet activation (76, 77).

In addition to Parkin, there are several other ubiquitin E3 ligases, such as SMURF1, SIAH1, Gp78 also involve in mitophagy regulation (78, 79). All these factors could generate ubiquitin chains after being located on the mitochondrial surface, followed by the recruitment of autophagy adaptors such as optineurin, nuclear dot protein 52, and p62. These adaptors interact directly with LC3, anchoring Ub-tagged mitochondria into autophagosomes. PINK1 ubiquitin kinase mediates optineurin, nuclear dot protein 52 recruitment on damaged mitochondria, stimulating mitophagy. The serine/threonine-protein kinase TBK1 modulates the phosphorylation status of the adaptors, followed by their increasing binding affinity to Ub chains, and promoting mitochondrial removal (80–82). Choong et al. (83) reported that damaged mitochondria could release into extracellular space in free naked form or in membrane-surrounded vesicles. Mitochondrial stress may enhance this extracellular release process. Extracellular mitochondrial release acts as an alternative pathway to PRKN-dependent and independent mitophagy to help with the clearance of damaged mitochondria.

Macrophages are the most heterogeneous immune cell population, which could be activated by a variety of cytokines. A recent work examining macrophage transcriptome in the mice heart post- MI showed a robust reprogramming of mitochondrial genes, suggesting that mitochondrial function may lie at the heart of macrophage function and cardiac remodelling. Another primary function of macrophages is to eliminate unwanted material through phagocytosis (84). Nicolás-Ávila et al. (85) identified a non-canonical route of elimination of abnormal mitochondria from cardiomyocytes in vesiculated structures (exophers). The exophers is then taken up and processed by macrophages surround cardiomyocytes through the phagocytic receptor Mertk. In cardiac stress such as AMI and hypertrophy, failure to eliminate mitochondria-laden exophers results in activation of the inflammasome and autophagy arrest, ultimately compromising mitochondrial fitness.

Cardiac remodelling is also a canonical pathological process after myocardial infarction, characterised with a large number of cardiomyocytes undergoing cell death. To maintain normal cardiac output, surviving cardiomyocytes will increase in cellular size, mass, and volume. In addition to cardiomyocytes, cardiac fibroblasts are also activated to secret components of the ECM, which promotes the differentiation to myofibroblasts and exert increased migratory, proliferative and secretory properties (86). BNIP3 is an apoptosis-inducing protein, Diwan et al. (87) and Dorn et al. (88) reported that BNIP3 ablation in unstressed mice reveals no essential function, but BNIP3 specific knockout mice suffered reduced apoptosis and cardiac remodelling after myocardial infarction. It is possible that BNIP3 regulates mitochondrial quality through mitophagy under baseline conditions. However, during cardiac injury, BNIP3 may act as a death promoter. Pingjun and colleagues found that RIPK3 mediated cardiomyocyte necroptosis via AMPK/Parkin-mitophagy axis in post-MI heart failure (89). Moshi et al. (90) found that conditional ablation of Drp1 in mouse embryonic fibroblasts promoted mPTP-mediated mitophagy. In summary, mitophagy is indispensable for physiological mitochondrial function, interruption of which may reduce mitochondrial dysfunction both in ischemia and post-ischemic reperfusion.

## Mitochondrial ROS in Ischemic Heart Disease

**Figure 4:**
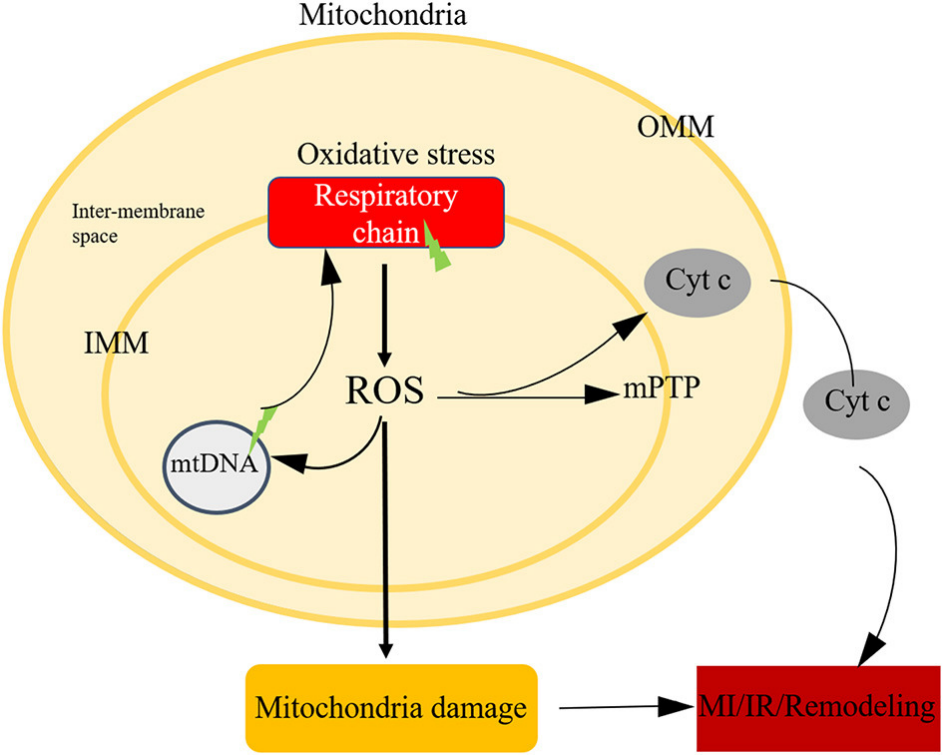
Mitochondria oxidative stress in ischemic heart disease. Mitochondria is the main source of ROS, after oxidative stress, the respiratory chain could generate ROS, the imbalance level of ROS could attribute to mtDNA damage, initiate the open of mPTP, leading to cell death.

It's undoubtful that ROS is a toxic product of aerobic metabolism, involving in various physiological and pathological processes (91, 92). Mitochondria is the major cellular source of ROS. On the one hand, mitochondria consume more than 95% of the oxygen to generate the energy required to sustain life (93). During ischemia, oxygen transported to mitochondrial ETC reduced sharply, after blood restoration bring back oxygen, an electron back-up primer the soluble ubiquinone component of the ETC (especially complex I and complex III) to generate oxygen free radicals. On the other hand, NADPH oxidase was another source of ROS. NADPH oxidase could not deal with the superfluous ROS, causing damage to DNA, proteins, lipids or modulate cellular signalling pathways (Figure 4).

Increased ROS levels could activate various second message pathways, such as the ERK, P38, protein kinase C, and PI3-kinase pathways (94–96). Zorov et al. reported that mtROS could dissipate the mitochondrial membrane potential and lead to mPTP opening (97). In the following study, Aon et al. (98) demonstrated that during ischemia-reperfusion injury, excessive ROS from ETC could activate the inner mitochondrial anion channel, causing the release of ROS into the cytoplasm and simultaneous dissipation of the membrane potential. As mentioned above, Nox is another important source of ROS. Braunersreuther et al. reported that in Nox1/Nox2 knockout mice, myocardial infarct size was significantly smaller than that in wild-type mice subjected to IR (30 min of ischemia and 24 h of reperfusion), the underlying pathways include Akt/ERK in Nox1-knockout mice and STAT3/ERK in Nox2-knockout mice (99). Matsushima et al. (100) aimed to figure out the role of Nox-4 in mediating IR injury and they found that Nox-4 knockout mice suffered reduced ROS production and attenuation of the infarct size after IR via the HIF-1α/PPARα pathway.

In addition to the harmful aspects, redox signalling also contributes to protective or adaptive responses during IR injury (101, 102). Many potential signal pathways have been reported in this process, HIF signalling is one of the most important ones. During ischemia, cardiomyocyte energy metabolism switches from FAO to glycolysis, under control of HIF, followed by the activated expression of several glycolytic genes (103–105). In addition, it's reported that oxidative stress following IR injury is neutralised by the CNC -bZIP transcription factor Nrf2, which could regulate intracellular redox homeostasis. With the accumulation of intracellular oxidants, the levels of Nrf2 increased in the nucleus, binding to ARE in the upstream regulatory regions of genes encoding detoxification and antioxidant enzymes, enhancing their transcription. This has been shown to protect the heart from IR injury (106, 107). Recently, some studies investigated that MAOs, including MAO-A and MAO-B, is another source of ROS. Located on the OMM, MAOs could generate O_2_ and H_2_O_2_ (108). During IR injury, the increased activity of the MAO-A isoform significantly deteriorated myocardial injury (109, 110) and promoted the cardiac remodelling (110).

Cardiac fibrosis is a significant feature of adverse cardiac remodelling after myocardial infarction, sustained fibrosis could result in myocardial stiffness, decrease of heart function, and increased risk of arrhythmias (111). After myocardial infarction, cardiac fibroblasts transform to a proinflammatory state, secreting cytokines and MMPs, later post-MI phase, fibroblasts transform to anti-inflammatory phenotype and generate ECM (112). It's reported that ROS is an important regulator of MMPs, the increased levels of ROS could increase the activity of MMPs, decrease tissue inhibitors of MMPs and increase collagen synthesis (113, 114). *In vitro* tests indicated that ROS could decrease collagen synthesis and increase transcriptional and posttranslational levels of MMPs (115, 116). *In vivo*, mice receiving ROS scavengers after MI could preserve left ventricular function via decreasing the activity of MMPs (117, 118). The evidence indicated that MMPs are key regulators in the process that ROS influences cardiac fibrosis. Gpx is an antioxidant enzyme, which could scavenge H_2_O_2_, meanwhile prevent the formation of other kinds of toxic radicals. Gpx transgenic mice presented improved heart function via attenuating apoptosis, fibrosis, and decreasing MMP-9 activation after MI (119). Some studies also identified the effect of mitochondrial oxidative stress on remote myocardium after MI. Overexpression of Prx3, a mitochondrial antioxidant enzyme, could inhibit cardiac remodelling and failure (120). Cardiolipin inhibition could prevent adverse cardiac remodelling in the non-infarcted MI border zone via the restoration of ETC and reduced ROS (121). In addition, another study demonstrated that increased lipid peroxidation products could be detected in post-MI heart failure (122). These studies indicated that mitochondrial oxidative stress is an important factor regulating ischemic heart disease.

## Mitochondria-Related Inflammation in Ischemic Heart Disease

**Figure 5:**
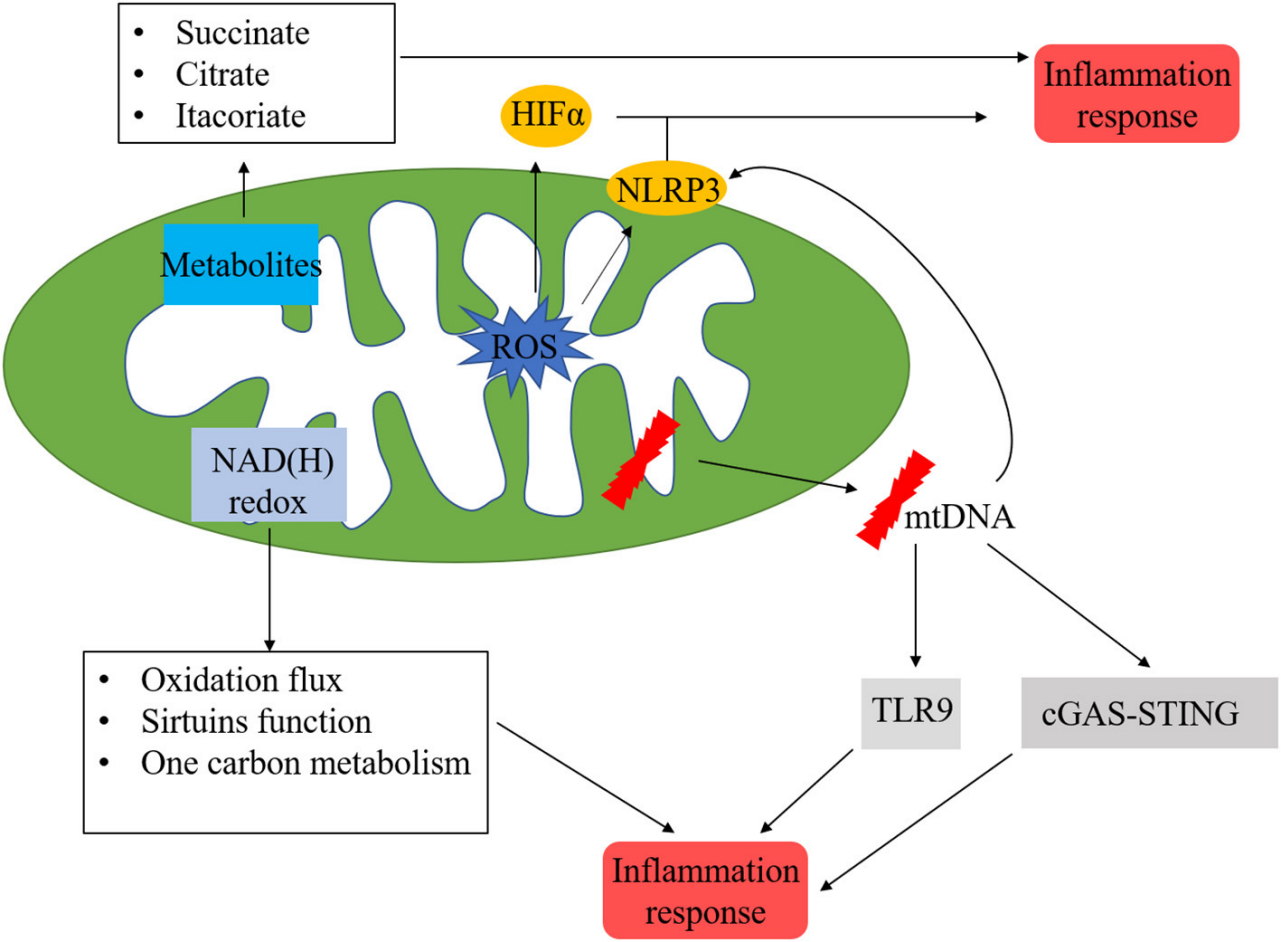
Mitochondria-related inflammation in ischemic heart disease. Many factors in mitochondria could initiate mitochondria-related inflammation, ROS from mitochondrial respiratory chain could directly activate HIFα and NLRP3, which are vital regulators in inflammation process. In addition, metabolites from mitochondria, such as succinate, citrate, and itacoriate also cause inflammation. mtROS could lead to mtDNA damage, which trigger the inflammation response via NLRP3, cGAS/STING, and TLR9 pathways. NAD(H) redox is another initiator of inflammation response via the regulation of oxidation flux, sirtuins function, and one carbon metabolism.

Current evidence demonstrated that inflammatory cell recruitment, together with the activation of innate and adaptive immune reactions, are the features of MI and IR injury (123, 124). Inflammation is an important component of tissue repair. However, recent studies suggested that excessive inflammation-related processes contributed to poor outcomes. In a steady-state, leukocytes and macrophages are the most prevalent subset in adult mouse hearts. After MI, B-, and T-cells were recruited to myocardium, leading to their increase of 5–10-folds. Circulating blood monocytes migrate into the infarcted heart and differentiate into macrophages. Many signalling pathways have been identified to be involved in mediating inflammation in acute and chronic myocardial injury. In this section, we mainly focused on the relationship between mitochondria and inflammation during cardiac injury (Figure 5).

The inflammasome is a cytoplasmic multiprotein complex that contributes to the release of mature cytokines during the innate immune response. Inflammasome could recognise PAMPs or host-derived DAMPs, recruiting, and activating the pro-inflammatory protease caspase-1. NLRP3 inflammasome consists of NLRP3, ASC, and caspase-1 proteins, which play important roles in the pathophysiology of MI. Mitochondrial events and NLRP3 inflammasome activation are tightly bounded. The NLRP3 inflammasome could induce mitochondrial damage via mtROS (125), decreased production of mtROS could effectively inhibit the activation of NLRP3 inflammasome (126). In addition, the insufficiency of damaged mitochondria clearance due to disturbed mitophagy flow would strengthen the activation of the NLRP3 inflammasome. mtDNA is a potential pro-inflammatory trigger for immune cells and is widely accepted as a member of DAMPs (127). Kiichi et al. (128) indicated that mtDNA released into cytoplasm might activate NLRP3 inflammasome, mitophagy could clear damaged mitochondria, followed by the inhibition of NLRP3 inflammasome. Depleting the autophagic proteins LC3B and beclin 1 increased the activation of caspase-1along with secretion of IL-1 β and IL-18. Calcium homeostasis is a critical factor for maintaining mitochondrial function. NLRP3 stimulators (such as ATP) may result in calcium inflow and lead to mitochondrial damage, followed by an increase of mtROS and depletion of mitochondrial membrane potential (129). cGAS/STING is another reported cytosolic mtDNA-sensing pathway, when mitochondria damage leads to the release of fragmented mtDNA into the cytosol, cGAS activates STING, followed by the activation of TBK1, resulting in the translation of interferon genes (130). The inflammatory process initiated by mtDNA is a critical mechanism of ischemic heart disease.

Emerging evidence suggests that macrophage function is closely associated with its mitochondrial metabolism (131, 132). Changes in mitochondrial function have been observed in activated macrophages. In pro-inflammatory macrophages, impairment of TCA flux leads to the accumulation of metabolic intermediates such as succinate and malate (133), overload of succinate is linked to abnormal ROS production (134). The generation of αKG from glutaminolysis is important for alternative M2 activation of macrophages via JMJD3-dependent epigenetic reprogramming of M2 genes (135). Shuang et al. (136) reported a new mechanism of macrophage in the process of myocardial repair after MI, in which efferocytosis increased the level of cellular fatty acids, the increased fatty acids fueled mitochondrial respiration and activated an NAD^+^-dependent signal transduction cascade, and this process is positive for wound healing. In 2015, Xu et al. (137) reported that the NOTCH signalling pathway is involved in mitochondrial metabolism remodelling, resulting in mtROS generation and pro-inflammatory gene expressions, such as TNF α and IL-1β.

There are other participants reported to be related to the mitochondria-related inflammation process. Mst1 is a stress-activated, pro-apoptotic kinase, Jing et al. (138) reported that the SRV2 deletion inactivated the Mst1-mROS signalling pathway in cardiomyocytes, which could regulate the inflammation and oxidative stress. Another regulator of inflammatory process-S100a8/a9 caused cell death in the early stage of IR injury via mitochondrial respiratory dysfunction (139). Mechanistically, S100a8/a9 downregulated NDUF gene expression which will inhibit the activity of mitochondrial complex I via Toll-like receptor 4/Erk–mediated Pparg coactivator 1 alpha/nuclear respiratory factor 1 signalling suppression.

## Mitochondrial Protein Post-Translational Modification in Ischemic Heart Disease

PTMs are alterations of proteins occurring after the translational process catalysed by numerous enzymes. Protein PTMs are important in various physiological and cellular processes, such as differentiation (140), protein degradation (141), gene expression (142, 143). PTMs of proteins have been identified to affect mitochondrial quality control, leading to the exacerbation, or alleviation of ischemic heart disease (144–146) (Figure 2).

Protein phosphorylation plays a critical regulatory role in cardiomyocytes via mediating protein activation or deactivation. Phosphorylation of mitochondrial proteins is vital to maintain mitochondrial function. Phosphorylation of mitochondrial complex IV subunit mediated its physical function in myocardial mitochondria. During IR injury, protein kinase A-dependent phosphorylation of complex IV increased and resulted in a decrease of its activity, followed by an increase in ROS production (147, 148). The STAT3 is a key regulator of mitochondrial metabolism via the interaction with mitochondrial proteins. In ischemic conditions, STAT3 phosphorylation improved mitochondrial function via preserving mitochondrial complex I, preventing mPTP opening with the result of infarction area reduction (149–151).

In eukaryotic cells, the UPS is a primary system of protein degradation. The ubiquitination occurred via ubiquitin-binding its COOH group with the target protein (152, 153). Ubiquitination has been widely accepted as one of the major ways of protein degradation to maintain mitochondrial quality. As we presented previously, PINK/Parkin-mediated mitophagy is a critical pathway for mitochondrial quality control. Parkin is an E3 ligase, which can ubiquitinate several mitochondrial outer membrane proteins via E3 ligase activity to recruit the p62 protein. CypD ubiquitinated by Parkin could inhibit mPTP opening, alleviating myocardial injury (154). In the process of heart remodelling after IR injury, exogenous ubiquitin supplement could reduce caspase-9 expression in the mitochondrial death pathway, increasing mitochondrial production, reducing infarct area, and finally restoring heart function (155). Wangxing et al. (156) demonstrated that leptin-overexpressing hMSCs into the infarcted heart could improve cardiac function. Further mechanical exploration indicated that leptin restored mitochondrial respiratory function via enhancing OPA1 expression by inhibiting the activity of OMA1, a mitochondrial protease. In addition, phosphorylation of GSK3 is a prerequisite for ubiquitination-depended degradation of OMA1 and attenuation of long-OPA1 cleavage.

SUMO is a member of the large family of ubiquitin-like proteins. SUMOylation is a classic ubiquitination-like PTMs, linking the SUMO protein to the lysine residue of the substrate protein (157). In heart IR injury, the binding of SUMO with Drp1 increased to form a complex, increasing the acidification of the complex could maintain mitochondrial quality and improve cardiac function (158). Another deSUMOylation-related enzyme, SENP3 could alleviate IR injury via the inhibition of Drp1 localisation in the mitochondria (159). Acetylation is one of the major PTMs in cell biology, SIRT3 and SIRT5 are sirtuins found in mitochondria. Angela et al. reported that SIRT3 could reduce the activity of CypD, inhibiting the opening of mPTP. Increasing SIRT3 expression in the failing heart could improve cardiac function (160). Studies showed that decreased expression of SIRT3 in heart increased susceptibility to IR injury (161, 162). In addition, SIRT1 was also found to be involved in cardiac IR injury, study found that increased expression of SIRT1 could restore left ventricular function during the construction of myocardial IR models (163). Zhao et al. invested the role of HDACs inhibition in myocardial IR injury and found that HDACs inhibition protected the heart against I/R injury (164). Moreover, SIRT5 (–/–) mouse hearts are more tendentious to suffer IRI due to the increase of lysine succinylation followed by the accumulation of mitochondrial ROS, and mtROS scavenged by SDH inhibition could reverse this process (165). Accordingly, the exploration of the interaction of PTMs in ischemic heart disease by modulating mitochondrial quality control has a bright future to investigate novel therapeutic targets.

MiRNAs are short non-coding RNA binding to the 3' UTR sequences and regulating targeted gene expression either by mRNA degradation or translational repression (166). Mitochondria contain miRNAs that are termed as mitomiRs in several species and cell types (167). MitomiRs target either mitochondrial or nuclear proteincoding mRNAs, thereby influencing mitochondrial metabolism and dynamics through regulation of the main mitochondrial pathways, such as OXPHOS, ETC components, TCA cycle (168). Overexpression of miRNA-34a in AMI patients' serum enhances cardiomyocyte apoptosis by down-regulating mitochondrial anti-apoptotic protein aldehyde dehydrogenase 2 (169). miRNA-1 could enter mitochondria and regulate mitochondrial ETC via targeting proteins in ETC networks, while increased miRNA-1 expression has been found in the remote myocardium of AMI patients (170). Mitochondrial miRNA-762 regulates cardiomyocyte apoptosis via impairing the core subunit of mitochondrial complex I (171). In addition, lncRNAs are also a heterogeneous class of transcripts involved in the epigenetic regulation of gene and genome activity (172, 173). Recent data indicated that lncRNAs localised to mitochondria, regulating mitochondrial function (174–176). Circulating levels of mitochondrial lncRNA LIPCAR were downregulated early after AMI and upregulated during later stages and were associated with adverse cardiac remodelling and death (175). In addition to lncRNAs, growing evidence also reports that circRNAs are involved in the regulation of the mitochondrial dynamics and cardiomyocyte apoptosis. Kun et al. (177) found that MFACR regulated mitochondrial fission and apoptosis in the heart by targeting and downregulation of miR-652-3p. To sum up, mitochondrial non-coding RNAs are involved in the pathogenesis of myocardial infarction via regulating various pathways.

## Targeted Therapies

Early reperfusion of occluded coronary arteries is the most effective strategy of AMI over the past decades. However, there is no effective therapy for reperfusion injury and alleviation of cardiac remodellin. Therefore, with a growing understanding of the molecular mechanisms of ischemia, IR injury, and chronic remodelling, we may develop more novel therapeutic targets to protect the heart from IHD and improve clinical outcomes of these patients. Considering numerous studies in this field, we only discussed the agents involved in mitochondrial targets in animal studies or clinical trials.

Over decades, mtROS is one of the most popular targets of heart protection. A reduced generation or increased scavenge of mtROS have been reported to increase outcomes. MitoQ is the first mitochondria-targeted antioxidant, which is bioavailable orally without toxicity detected. Rat received MitoQ for 2 weeks suffered reduced oxidative stress and resisted heart ischemia-reperfusion injury (178). SS-31 is a kind of small artificial peptides with therapeutic potential due to its antioxidant properties (179–181), in rat tests, SS-31 could attenuate the strike of ischemia and reperfusion via reducing MI size (180, 182, 183). SODs are metal-containing antioxidant enzymes, protecting cells from damage by converting superoxide radicals to H_2_O_2_ and O_2_. Mito-specific SOD mimetics exert protection under oxidative stress (184). Clinical trials of SS-31 in patients with heart failure and acute myocardial infarction have been tested, although the myocardial infarct size did not show an improvement, it showed acceptable safety and tolerability (185, 186). Cerrato's group two analogues of SS-31 (mtCPP-1 and mtgCPP) and reported greater efficiency and antioxidant capacity than SS-31 (187, 188). In an animal model of post-infarction heart failure, activation of ALDH2 with Alda-1 improves the clinical outcomes via the decrease of reactive aldehydes (189).

mPTP is a mitochondria voltage- and Ca2+-dependent high-conductance channel. Piot et al. exerted a clinical trial that included 58 patients who suffered acute ST-elevation myocardial infarction to receive cyclosporine immediately before undergoing PCI, it came out that cyclosporine could decrease the infarct size to some extent (190). Currently, some larger clinical trials are ongoing to test the effect of mPTP inhibition on short- and long-term patients' outcomes. Trehalose is a small molecule from mushrooms, which could activate mitophagy. Studies indicated that administration of trehalose for 4 weeks could reverse cardiac remodelling and fibrosis in MI model mice (191, 192). Spermidine is another natural compound that activates autophagy. In the MI rat model, oral supplementation of spermidine is inversely associated with all-cause mortality and MI risk via enhancement of mitochondrial respiration (193). In addition, Jing et al. reported that spermidine supplement improved MI-induced cardiac dysfunction through AMPK/mTOR mediated autophagic flux (194). Mdivi-1 is an inhibitor of mitochondria-related fission protein-Drp1, which is proved to reduce infarct size, rescue cardiac function in the IRI mouse model (195). In addition, mdivi-1 treatment ameliorated IRI via the inhibition of connexin 43 loss and suppression of MMP3 (196). UPR^mt^ is critical to maintain mitochondrial proteostasis under cellular stress, UPR^mt^ induced by oligomycin or doxycycline has been identified to reduce MI size in mice models (197). The expression of AMPK increased in failing heart, metformin is considered as a pharmacological activator of AMPK, which could reduce cardiac infarct size and improve heart contractibility in a rat MI model. Artificial compounds targeting AMPK have been designed, Abbott lab generated an artificial agent, named A769662, which could reduce infarct size in rats via specific activating AMPK on β subunit (198, 199).

Although various experimental studies developed effective cardioprotective strategies, there are few successful clinical translations. And this has been attributed to different factors: firstly, there are no effective delivery systems for agents to carry out their functions. Secondly, animal models are different from patients with ischemic heart disease, which usually also suffered from other co-morbidities such as hypertension, diabetes. Recently, some clinical agents such as DPP-4 and SGLT2 have been identified to protect cardiomyocytes from IRI damage via mitochondrial function preservation. GLP-1 is an incretin hormone with cardioprotective capacities and was markedly increased in acute myocardial infarction. In physical conditions, GLP-1 could be degraded by DPP-4, Sebastian et al. (200) found that DPP-4 inhibitor could maintain the serum concentration of GLP-1, increasing AMPK activity and mitochondrial respiratory capacity of non-infarcted tissues. In addition, GLP-1 agonist, liraglutide could reduce cardiac infarct size, protected cardiomyocytes from injury and preserved contractile function via suppressing ROS generation, NADPH oxidase and proinflammatory signals (201). SGLT-2 inhibitors are a new generation of anti-diabetic agents, which have been recommended in cardio-protection (202). Various studies proved that SGLT-2 inhibitors could ameliorate cardiac remodelling and increase mitochondrial function (203, 204). Another type of anti-diabetic agent, metformin is also reported to exert cardio-protection by restoring mitochondria function and dynamics in cardiac I/R injury (205). Some natural agents or analogues such as taurine, fisetin, and humanin also exert cardio-protection against IRI by reducing mitochondrial dysfunction (206–208). In summary, despite there is much evidence supporting the targeting of mitochondria as a therapy strategy in IHD, more efforts are needed to promote the basic to clinical translation.

## Conclusions

Mitochondrial homeostasis is critical for the maintain of the mitochondria network. In this review, we summarised the advances supporting the view that mitochondrial disorder is a major contributor to cardiac injury, IRI, as well as chronic remodelling. Mitophagy disorder, increased mtROS, mitochondria-related inflammation and post-translation of mitochondrial proteins are considered contributory factors to mitochondrial dysfunction in ischemic heart disease. Accordingly, many targeted modulations involved in mitochondrial quality control provide great chances for the design of novel therapies. Although there are no drugs with successful clinical transformation, which are directly targeted mitochondria-related mechanisms, many regulatory proteins or peptides and miRNAs possess significant potential.

## Author Contributions

YX, JinL, HG, and JuL contributed to the first and second version of the manuscript. XZ and HL contributed to the second version. HL supervised the project. JiaL made great contribution to our revision manuscript and provided helpful opinions. All authors contributed to the article and approved the submitted version.

## Funding

This work was supported by Beijing Natural Science Foundation (No.7214219); Natural Science Foundation of Liao Ning Province (2019-MS-386).

## Conflict of Interest

The authors declare that the research was conducted in the absence of any commercial or financial relationships that could be construed as a potential conflict of interest.

## Publisher's Note

All claims expressed in this article are solely those of the authors and do not necessarily represent those of their affiliated organizations, or those of the publisher, the editors and the reviewers. Any product that may be evaluated in this article, or claim that may be made by its manufacturer, is not guaranteed or endorsed by the publisher.

## Glossary

ALDH2, Aldehyde dehydrogenase 2; ANT, adenosine nucleotide translocase; AMPK, Adenosine 5′-monophosphate (AMP)-activated protein kinase; AMI, Acute Myocardial Infarction; ARE, Antioxidant response elements; ASC, Apoptosis-associated speck-like protein; α-KG, α-ketoglutarate; ATP, Adenosine triphosphate; BNIP3, BCL2 Interacting Protein 3; cGAS, GMP-AMP synthase; circRNA, covalently closed, single-stranded RNA; CNC, Cap “n” collar; CREB, cAMP-response element binding protein; CypD, Cyclophilin D; DAMP3, Danger signalling molecules 3; DPP4, Dipeptidyl peptidase-4; Drp1, Dynamin-related protein1; ECM, Extracellular matrix; ETC, electron transport chain; ERK, Extracellular signal-regulated kinas; FAO, Fatty acid β-oxidation; FUNDC1, FUN14 Domain Containing 1; GLP1, Glucagon-like peptide 1; Gpx, Glutathione peroxidase; GSK3, Glycogen synthase kinase-3; GTPase, Guanosine triphosphatases; HDAC, Histone deacetylases; HF, Heart failure; HIF, Hypoxia-inducible factor; hMSC, Human mesenchymal stem cells; IHD, Ischemic heart disease; IL-1β, Interleukin-1β; IRI, Ischemia reperfusion injury; LC3, Microtubule- associated protein1 light chain 3; lncRNA, Long noncoding RNAs; MAO, Monoamine oxidases; MAPK, Mitogen - activated protein kinase; MCU, Mitochondrial Calcium Uniporter; MFACR, Mitochondrial fission and apoptosis-related circRNA; Mfn1/2, Mitofusin 1/2; miRNA, MicroRNA; mitomiRs, Mitochondria miRNAs; MMP, Matrix processing peptidases; MPP, Matrix metalloproteinases; mPTP, Mitochondrial permeability transition pore; mRNA, Messenger RNA; Mst1, Mammalian STE20-like protein kinase 1; mTOR, Mammalian target of rapamycin; NAPDH, Nicotinamide adenine dinucleotide phosphate; NLRP3, Nucleotide-binding oligomerization domain-like receptor family pyrin domain containing 3; Nox, NADPH oxidases; NR4A1, Nuclear receptor subfamily 4 group A member 1; OMM, Outer mitochondrial membrane; OPA, Optica atrophy protein 1; OXPHOS, Oxidative phosphorylation; PAMP, Pathogen-associated molecular patterns; PCI, Percutaneous coronary intervention; PINK, PTEN-induced putative kinase 1; PTM, Post-translational modification; RIPK3, Receptor-interacting protein 3; ROS, Reactive oxygen species; SDH, Succinate dehydrogenase; SENP3, Sentrin-specific protease 3; SGLT2, Sodium-glucose cotransporter 2; SIRT, Sirtuin; SIAH1, Siah E3 ubiquitin protein ligase 1; SMURF1, Smad ubiquitination regulatory factor-1; SOD, Superoxide dismutase; SRV, Suppressor of ras val-2; STAT3, Signal transducer and activator of transcription 3; STING, Stimulator of interferon genes; SUMO, Small ubiquitin-like modifier; TBK1, TANK-binding kinase 1; TCA, Tricarboxylic acid cycle; TNF-α, Tumour necrosis factor α; UPR^mt^, Mitochondrial unfolded protein response; UPS, Ubiquitin-proteasome system; UTR, untranslated region; VDAC1, Voltage-dependent anion-selective channel protein 1; WDR26, WD repeat domain 26.
